# γ-H2AX + CD8+ T lymphocytes cannot respond to IFN-α, IL-2 or IL-6 in chronic hepatitis C virus infection

**DOI:** 10.1016/j.jhep.2012.12.009

**Published:** 2013-05

**Authors:** Matthew Hoare, Arun Shankar, Meera Shah, Simon Rushbrook, William Gelson, Susan Davies, Arne Akbar, Graeme J.M. Alexander

**Affiliations:** 1 Department of Medicine, School of Clinical Medicine, University of Cambridge, Cambridge, UK; 2 Department of Pathology, University of Cambridge, Cambridge, UK; 3 Department of Immunology, University College London, London, UK

**Keywords:** HCV, Hepatitis C virus, DSB, double-stranded DNA breaks, ATM, ataxia telangiectasia-mutated, IFN-α, interferon-α, PBMC, peripheral blood mononuclear cells, DDR, DNA damage response, SOCS, suppressor of cytokine signalling, Hepatitis C, T-lymphocytes, Senescence, Interferon-alpha, γ-H2AX

## Abstract

**Background & Aims:**

Age is the dominant prognostic factor influencing the natural history of hepatitis C virus (HCV) infection and treatment response. Accelerated lymphocyte telomere shortening in HCV infection correlates with adverse clinical outcomes. Critical telomere shortening generates double-stranded DNA breaks (DSB) inducing the DNA damage response, leading to replicative senescence. The phenotype and function of CD8+ T lymphocytes and the *in vitro* response to IFN-α in relation to the DNA damage response were investigated in patients with chronic HCV infection.

**Methods:**

CD8+ T lymphocytes with DSB were identified by expression of γ-H2AX (Ser-139) in 134 HCV-exposed subjects and 27 controls. Telomere length was determined by flow-FISH; cytokine expression by intracellular cytokine staining; *in vitro* responses to IFN-α, IL-2 or IL-6 by phospho-STAT1 (Y701) or phospho-STAT5 (Y694) expression.

**Results:**

The proportion of circulating CD8 + γ-H2AX+ T lymphocytes rose with increasing fibrosis stage (*p = *0.0023). CD8 + γ-H2AX+ T lymphocytes were enriched in liver compared to blood (*p = *0.03). CD8 + γ-H2AX+ T lymphocytes demonstrated increased IFN-γ (*p = *0.02) and reduced IL-2 expression (*p = *0.02). CD8 + γ-H2AX+ T lymphocytes failed to phosphorylate STAT1 in response to IFN-α compared to unfractionated CD8+ T lymphocytes (*p* <0.0001). More widespread failure of Jak/Stat signalling in CD8 + γ-H2AX+ T lymphocytes was suggested by impaired phosphorylation of STAT1 with IL-6 (*p = *0.002) and STAT5 with IL-2 (*p = *0.0039) compared to unfractionated CD8+ T-lymphocytes.

**Conclusions:**

In chronic HCV infection, CD8 + γ-H2AX+ T lymphocytes are highly differentiated with shortened telomeres, are more frequent within the liver, are associated with severe fibrosis and fail to activate Jak/Stat pathways in response to IFN-α, IL-2 or IL-6, perhaps explaining treatment failure in those with severe fibrosis.

## Introduction

Several factors influence the risk of progressive liver injury in chronic hepatitis C virus (HCV) infection, including male sex and alcohol misuse [1], but increasing age has perhaps the strongest association with progressive fibrosis and treatment failure [2–4].

Healthy aging is associated with shortened telomeres in lymphoid and non-lymphoid cells [5,6]. Short telomeres are detected by the MRN protein complex including MRE11, NBS1 and RAD50 [7,8], leading to recruitment of ataxia telangiectasia-mutated (ATM) and ataxia telangiectasia and Rad3 related (ATR) serine/threonine kinases [8,9]. A number of nuclear targets are phosphorylated subsequently, including Histone 2 at serine 139 to form γ-H2AX [10,11]. γ-H2AX recruits further ATM complexes to the site of its formation in a positive feedback loop and also initiates stabilisation of p53 and its downstream target p21, leading ultimately to cell-cycle arrest [10,12].

Cellular responses to interferon-α (IFN-α) are important following exposure to HCV and during anti-viral therapy. IFN-α binds to IFN-AR2, which forms a heterodimer with IFN-AR1, triggering an intracellular signalling cascade through the Jak/Stat pathway [13]. Phosphorylation of Jak1 and Tyk2 associated with the intracellular tail of IFN-AR1 leads to phosphorylation of STAT1 at tyrosine 701. Treatment outcome in HCV infected patients is related closely to the transcriptome generated in response to IFN-α [14–16].

HCV modulates cellular responses to endogenous IFN-α signalling through pSTAT1. HCV core prevents phosphorylation of STAT1 in response to IFN-α [17] and NS5A prevents translocation of pSTAT1 to the nucleus [18]. Shorter telomeres are associated with defective activation of STAT5 in mouse macrophages [19].

In a previous study in chronic HCV infection progressive fibrosis, an adverse clinical outcome and impaired treatment responses were each associated with shortened CD8+ and CD4+ T-lymphocyte telomeres [20]. We therefore investigated the *in vitro* response to IFN-α in CD8+ T cells in relation to shortened telomeres and γ-H2AX expression as a measure of double strand DNA break signalling.

## Materials and methods

### Subjects (Table 1)

Patients recruited at Addenbrooke’s Hospital, Cambridge gave written informed consent with approval of the Local Research Ethics Committee. Patients co-infected with HIV, HBV or with other chronic liver diseases, identified by history, blood tests or liver biopsy, were excluded. Lymphocytes from healthy controls were obtained from local volunteers; none gave a history of chronic illness or intravenous drug usage. Liver biopsies were scored according to Ishak criteria by a specialist liver histopathologist (SED). Fibrosis was staged 0 (absent)–6 (cirrhosis); mild liver disease was defined as a fibrosis score of 0–3 and severe 4–6.

### Viral serology and PCR

Routine serology and PCR were performed in the Department of Virology, Addenbrooke’s Hospital, Cambridge as described previously [20].

### Lymphocyte preparation, culture and T-cell receptor directed stimulation

Peripheral blood mononuclear cells (PBMCs) were obtained by centrifugation of citrated blood over Lymphoprep (Nycomed). Cells were cultured in RPMI-1640 medium supplemented with 2 mM l-glutamine, 10% foetal calf serum (Biosera), 100 IU/ml penicillin and 0.1 mg/ml streptomycin (Sigma–Aldrich).

Cells were cultured with or without variable concentrations of interferon-α2b (PBL biomedical laboratories), IL-2 or IL-6 (Roche Applied Sciences).

Control (unstimulated) PBMC or PBMC stimulated with 1 μg/ml plate-bound anti-CD3 and 4 μg/ml soluble anti-CD28 (BD) were incubated in a humidified 5% CO_2_ atmosphere for 15 h. After 2 h, Brefeldin A (BD) was added according to the manufacturer’s instructions.

### Liver biopsy

See Supplementary Materials and methods.

### Flow cytometry

All cytometry was performed on a FACSCanto II analyser (BD); data were analysed with FACSDiva software (BD).

PBMCs were stained for cell surface markers before fixation in CALTAG medium A. Cells were permeabilised in ice-cold 90% methanol (VWR) and then stained with combinations of Alexa Fluor (AF) 488 or AF647 conjugated anti-γ-H2AX (Ser-139) (Cell Signaling) or other intracellular antigens or appropriate isotype controls. For further details of antibodies see Supplementary Materials and methods.

A positive control for γ-H2AX (PBMCs irradiated with 50 Gy) was included in every run. Streptavidin-Cy3 (Cedarlane Labs) was utilised with biotinylated primary antibodies. Class 1 pentamer analysis was performed in HLA-A2 positive individuals utilising HCV NS3 (KLVALGINAV) or CMV pp65 (NLVPMVATV) according to the manufacturer’s instructions (Proimmune).

### Telomere length by flow cytometry

Telomere length of CD8+ T cells was measured using flow-FISH assay [20]. CD4+ PBMCs from one healthy individual were analysed in every experiment as an internal control. Lymphocyte telomere length within an individual was expressed as mean fluorescence intensity (MFI). CD8+ lymphocytes were negatively selected using the CD8+ isolation kit II (Miltenyi Biotec) prior to staining and *in situ* hybridisation. CD8+ purity always exceeded 90%.

### Immunoblotting

See Supplementary Materials and methods.

### Statistics

Data are the median (interquartile range), unless stated otherwise. γ-H2AX expression in different patient groups and cell surface phenotype of γ-H2AX+ cells were analysed by Kruskal–Wallis test. Paired data from unfractionated CD8+ and γ-H2AX + CD8+ T cells were analysed by Wilcoxon signed rank test. Absolute pSTAT1 responses and p53 expression were analysed by 2-way ANOVA. Dose–response curves and EC_50_ were calculated by Prism 5.0 (Graphpad Software). The correlation between γ-H2AX expression and telomere length, age or BMI was analysed by Spearman’s rank correlation.

Multiple linear regression analysis was performed to identify predictors of γ-H2AX expression using SPSS 15.0. Only variables with a *p* value of <0.10 on univariate analysis were subjected to multivariate analysis. *p* values of <0.05 were considered significant.

## Results

**Fig. 1:**
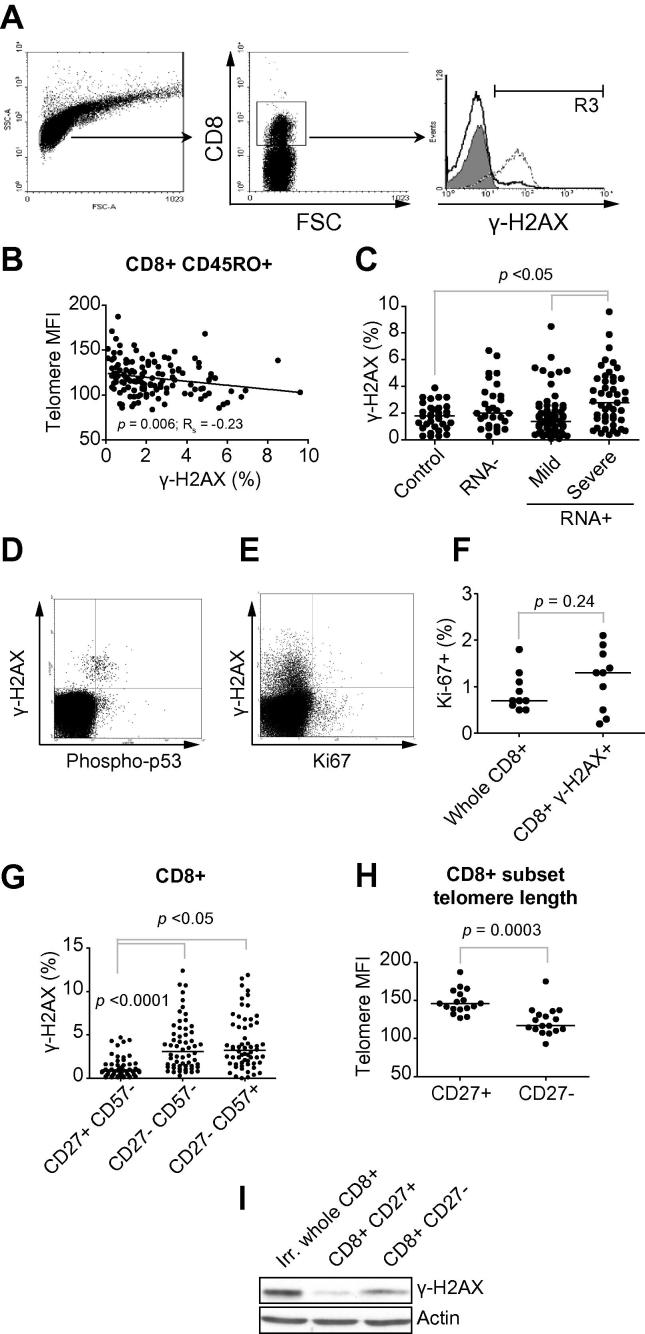
**γ-H2AX (Ser-139) expression on circulating CD8+ T lymphocytes in healthy controls and HCV-infected subjects**. (A) Gating strategy for γ-H2AX; cells within the live lymphocyte gate by scatter characteristics (left panel) and positive staining for CD8 (centre panel) were studied. Isotype control staining pattern in filled histogram; positive control staining pattern from irradiated cells in dashed histogram, and experimental sample in bold histogram (right panel). (B) Association between telomere length in CD8 + CD45RO+ subsets and γ-H2AX expression. Correlation by Spearman’s rank. (C) γ-H2AX levels in CD8+ T lymphocytes (27 controls, 27 HCV-RNA- HCV-exposed cases, 59 HCV-RNA+ patients with mild fibrosis and 48 HCV-RNA+ patients with severe fibrosis) by study group allocation. (D) Co-expression of γ-H2AX and phospho-p53 (Ser 15) in CD8+ T lymphocytes from subjects with viraemic HCV infection. (E and F) Co-expression of γ-H2AX and Ki67 in CD8+ T lymphocytes from 10 HCV-RNA+ subjects. Analysis by Wilcoxon signed rank test. (G) Cell-surface phenotype of circulating γ-H2AX + CD8+ T lymphocytes from subjects (n = 60) with HCV infection. CD8+ lymphocytes were divided based on the expression of the surface markers CD27 and CD57. Statistical analysis by Friedman test with Dunn’s multiple comparison test. (H) CD8+ T lymphocyte telomere length from CD8+ subsets based on CD27 expression from 10 HCV-RNA+ subjects. (I) Immunoblot of magnetic-bead separated CD8 + CD27+ and CD27- cells for γ-H2AX and β-actin. Irradiated whole CD8+ lymphocytes were a positive control for γ-H2AX expression.

Flow-cytometry was used to detect γ-H2AX, a marker of DNA double-strand breaks, to investigate the relation between peripheral CD8+ T-lymphocyte telomere length and downstream signalling (Fig. 1A). Subjects with CD8 + CD45RO+ T lymphocytes with shortened telomeres had an increased proportion of circulating γ-H2AX + CD8+ T lymphocytes (*p *= 0.006, Rs = −0.23, Fig. 1B). The proportion of circulating γ-H2AX + CD8+ T lymphocytes in HCV-RNA+ patients with severe fibrosis (median 2.8% (1.5–4.6)) was higher than in healthy controls (1.8% (0.98–2.4)), HCV-RNA-negative HCV-exposed subjects (2.0% (1.1–3.5)) and HCV-RNA-positive patients with mild fibrosis (1.4% (0.8–2.4), Kruskal Wallis *p *= 0.0023, Fig. 1C).

To study the independent association between clinical and demographic factors with CD8+ T-lymphocyte γ-H2AX expression in HCV-RNA+ subjects (n = 109), a multiple linear regression model was constructed (Supplementary Fig. 1 and Table 2). By univariate analysis, increased fibrosis stage (*p* <0.001) and a low ALT (*p *= 0.059) were associated with increased γ-H2AX expression. On multivariate analysis, increased fibrosis stage (*p* <0.001) and low ALT (*p *= 0.025) were independently associated with increased γ-H2AX expression (Table 2).

There was increased γ-H2AX expression on CD8+ T lymphocytes in subjects with past exposure to CMV (Supplementary Fig. 1G–I), which is probably explained by a higher proportion of CD8+ T lymphocytes with an advanced cell surface phenotype, defined by CD27 and CD57 expression and described previously in CMV [21] (Supplementary Fig. 1H). There was no increase in γ-H2AX expression when the analysis was restricted to the CD27- subset, controlling for this increase in highly differentiated cells (Supplementary Fig. 1I).

γ-H2AX expression was associated with evidence of downstream DNA damage response (DDR) signalling, as γ-H2AX + CD8+ T lymphocytes had increased phosphorylation of p53 at serine 15 (Fig. 1D). There was no difference in Ki67 expression between unfractionated CD8+ T lymphocytes (0.7%; 0.57–1.15) and γ-H2AX + CD8+ T lymphocytes (1.3%; 0.45–1.75, *p *= 0.24, Fig. 1E and F), indicating γ-H2AX expression was unrelated to DNA replication.

γ-H2AX was present in a higher proportion of CD8+ T lymphocytes expressing the mature phenotypes CD27-CD57- (3.1% (1.4–5.6)) or CD27-CD57+ (3.2% (1.7–6.2)) than the less mature CD27 + CD57- subset (0.8% (0.4–1.6), *p* <0.0001, Fig. 1G). Consistent with this observation, CD8 + CD27- T lymphocytes had shorter telomeres (117 (111 – 134)) than CD8 + CD27+ T lymphocytes (146 (139–161), *p *= 0.0003, Fig. 1H). Immunoblotting revealed increased γ-H2AX expression in CD8 + CD27- T lymphocytes compared to CD8+ CD27+ T lymphocytes (Fig. 1I).

**Fig. 2:**
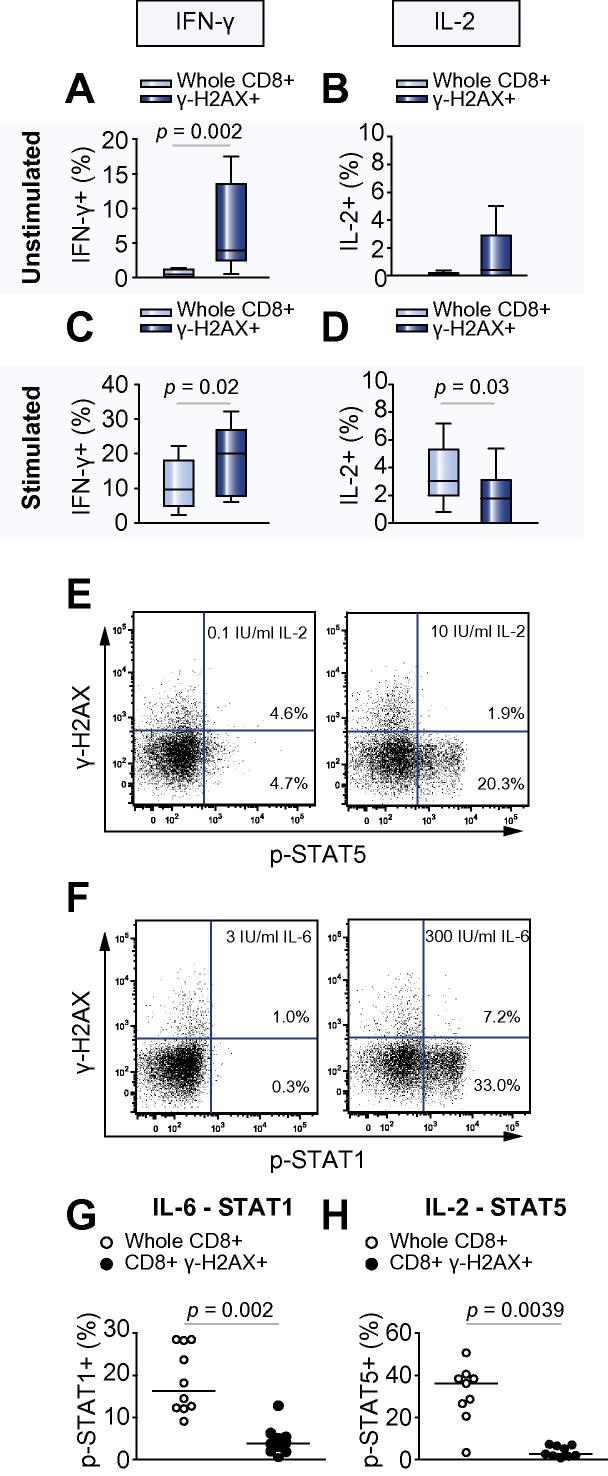
**Cytokine expression pattern of peripheral CD8+ T lymphocytes from 10 subjects with chronic HCV infection**. Unfractionated CD8+ lymphocytes or γ-H2AX + CD8+ subsets were analysed for expression of (A and C) IFN-γ, and (B and D) IL-2. Cells were either (A and B) unstimulated or (C and D) stimulated with anti-CD3/anti-CD28 overnight. Analysis by Wilcoxon signed rank test. Failure of several Jak/Stat signalling pathways in γ-H2AX + CD8+ T lymphocytes from HCV-RNA+ subjects (n = 10). Circulating T lymphocytes were incubated with IL-2 or IL-6 before staining for CD8, γ-H2AX and pSTAT1 (Tyr 701) or pSTAT5 (Tyr 694). Example dot-plots of CD8+ gated T lymphocytes incubated with (E) 0.1 or 10 IU/ml IL-2 or (F) 3 or 300 IU/ml IL-6. Panels G and H demonstrate the proportion of whole CD8+ and γ-H2AX + CD8+ T lymphocytes expressing (G) IL-6-induced phospho-STAT1 and (H) IL-2 induced phospho-STAT5. Analysis by Wilcoxon signed rank test.

Without stimulation, a higher proportion of γ-H2AX + CD8+ T lymphocytes expressed IFN-γ (3.9% (2.48–13.5)) compared with unfractionated CD8+ T lymphocytes (0.4% (0.08–1.2), *p *= 0.002, Fig. 2A). Following overnight stimulation (with anti-CD3 and anti-CD28), a higher proportion of γ-H2AX + CD8+ T lymphocytes expressed IFN-γ (20.0% (7.8–26.9) compared with 9.6% (4.9–18.1), *p *= 0.02) than unfractionated CD8+ T lymphocytes (Fig. 2C). However, the proportion of unstimulated γ-H2AX + CD8+ T cells expressing IL-2 was lower than unfractionated CD8+ T lymphocytes (1.8% (0–3.1) compared with 3.1% (2–5.3), *p *= 0.03, Fig. 2D).

We studied co-expression of γ-H2AX and markers of activation and known inhibitory receptors. γ-H2AX + CD8+ T cells were CD38 positive, but CD69 negative (Supplementary Fig. 2). Further, these cells did not express the inhibitory receptors PD-1 or Tim3 (Supplementary Fig. 2). γ-H2AX + CD8+ T cells were negative for CD161 [22] in 15 further patients with viraemic HCV infection (data not shown). To study potential antigen-specificity, circulating γ-H2AX + CD8+ T lymphocytes derived from three HLA-2 positive patients with positive CMV serology and current HCV infection (HCV-RNA+ in serum) showed no specificity for either HCV-NS3 or CMV-pp65, although unfractionated CD8+ T lymphocytes responded to both pentamers (Supplementary Fig. 3).

**Fig. 3:**
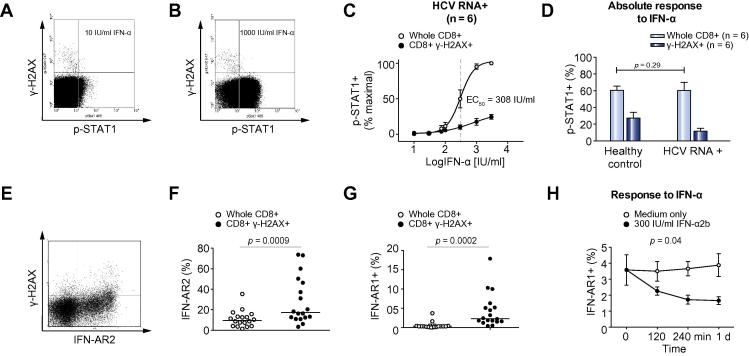
**CD8+ γ-H2AX+ lymphocytes fail to phosphorylate STAT1 after incubation with IFN-α unrelated to IFN-α receptor function**. Circulating T lymphocytes were incubated with variable concentrations of IFN-α2b before staining for CD8, γ-H2AX and pSTAT1 (Tyr 701). Example dot-plots of CD8+ gated T lymphocytes incubated with (A) 10 IU/ml or (B) 1000 IU/ml IFN-α2b. (C) Dose response curves of CD8+ and CD8 + γ-H2AX+ pSTAT1 responses by proportion of pSTAT1 positive cells after incubation with IFN-α in HCV-infected subjects (n = 6). (D) Maximal absolute pSTAT1 response to IFN-α in healthy controls (n = 6) and HCV-infected subjects (n = 6) in different CD8+ lymphocyte subsets defined by γ-H2AX. Analysis by 2-way ANOVA. Failure of CD8 + γ-H2AX + cells to phosphorylate STAT1 does not relate to STAT1 expression or IFN-α receptor components. (E and F) Expression of IFN-AR2 on whole CD8+ and γ-H2AX + CD8+ lymphocytes from HCV-RNA+ subjects (n = 18); (E) example cytometric data of IFN-AR2 and γ-H2AX co-expression; (F) IFN-AR2 expression in whole and γ-H2AX + CD8+ lymphocytes. (G and H). Expression and downregulation of IFN-AR1 on whole CD8+ and γ-H2AX + CD8+ lymphocytes from viraemic HCV infected subjects (n = 18); (G) IFN-AR1 expression in whole and γ-H2AX+ lymphocytes; (H) time course of IFN-AR1 expression in γ-H2AX + CD8+ lymphocytes from HCV-RNA+ subjects (n = 6) cultured in medium alone or with 300 IU/ml IFN-α2b for 24 h. Analysis by 2-way ANOVA.

Induction of pSTAT1 expression was used as a measure of the response to *in vitro* IFN-α (Fig. 3A and B). CD8+ T lymphocytes from healthy controls responded with an EC50 of 99 IU/ml. Unfractionated CD8+ T lymphocytes from HCV-infected subjects had an increased EC50 when compared to healthy controls (308 IU/ml), which was still higher in γ-H2AX + CD8+ T lymphocytes (781 IU/ml) (Fig. 3A–C). The maximal response of unfractionated CD8+ T lymphocytes from HCV infected patients was similar to that in healthy controls (*p *= 0.29, Fig. 3D) but the response of γ-H2AX+ T lymphocytes was impaired by comparison (*p* <0.0001). These defects could not be explained by total STAT1 content (Supplementary Fig. 4A and B), surface expression of IFN-AR components (Fig. 3E-G), failure to phosphorylate Tyk2 or Jak1 (Supplementary Fig. 4C and D) or IFN-α induced downregulation of IFN-AR from the cell surface of γ-H2AX + CD8+ T lymphocytes (Fig. 3H).

**Fig. 4:**
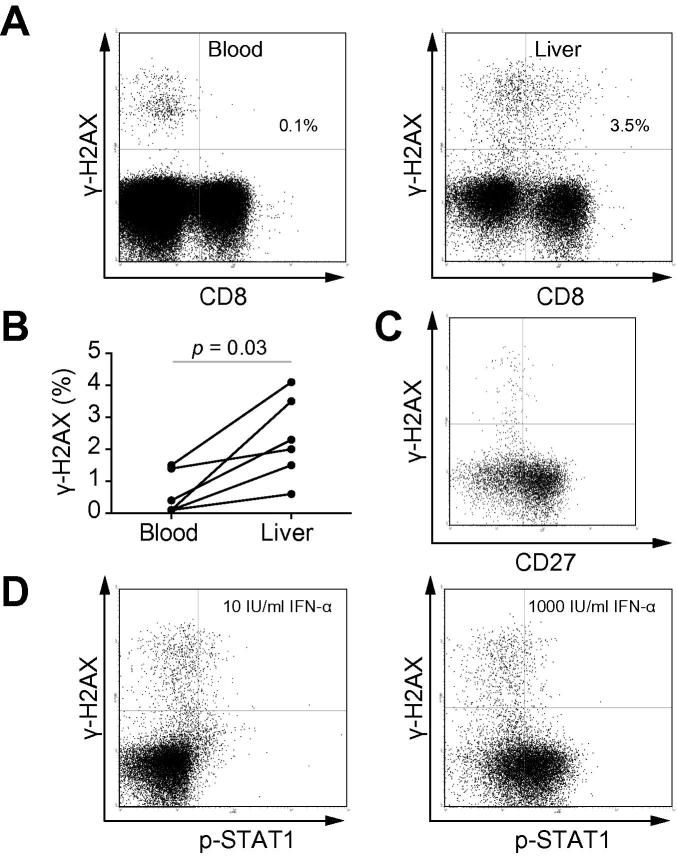
**CD8+ γ-H2AX+ T lymphocytes are more frequent within the liver of HCV-infected subjects**. (A) Example plots of CD8 and γ-H2AX expression on peripheral (left panel) and intrahepatic (right panel) lymphocytes from an HCV-RNA+ subject. (B) Expression of γ-H2AX on circulating and intrahepatic CD8+ T lymphocytes from 6 HCV infected subjects. Analysis by Wilcoxon signed rank test. (C) Co-expression of CD27 and γ-H2AX on intrahepatic CD8+ T lymphocytes. (D) Response of hepatic CD8+ T lymphocytes to *in vitro* IFN-α through STAT1 phosphorylation. Example dot-plots of hepatic CD8+ T lymphocytes incubated with 10 IU/ml (left panel) or 1000 IU/ml IFN-α2b (right panel).

A higher proportion of intrahepatic CD8+ T lymphocytes expressed γ-H2AX (2.15%, (1.28–3.65)) compared to circulating CD8+ T lymphocytes simultaneously isolated (0.25% (0.1–1.43), *p *= 0.03, Fig. 4A and B). Intrahepatic γ-H2AX + CD8+ T lymphocytes had a similar phenotype to circulating T lymphocytes with low expression of CD27 (Fig. 4C). Intrahepatic γ-H2AX + CD8+ T lymphocytes also failed to phosphorylate STAT1 in response to IFN-α2b (Fig. 4D).

To investigate whether the defect in Jak/Stat signalling was confined to the IFN-α pathway, the response of γ-H2AX + CD8+ T lymphocytes to IL-6 was investigated, as this also signals through STAT1. The proportion of unfractionated CD8+ T lymphocytes that expressed pSTAT1 after exposure to 300 IU/ml IL-6 was higher than in γ-H2AX + CD8+ T lymphocytes (16.4% (12.3–28.4 and 3.9% (1.9–5.6), *p *= 0.002, Fig. 2E and G) suggesting that defective STAT1 phosphorylation was not confined to IFN-α signalling.

IL-2-induced STAT5 phosphorylation was investigated to determine if there was an overall failure of Stat activation in γ-H2AX + CD8+ T-lymphocytes. After exposure to 10 IU/ml IL-2 the proportion of unfractionated CD8+ T lymphocytes that expressed pSTAT5 was higher than in γ-H2AX + CD8+ T lymphocytes (36.2% (23.6–39.5) and 2.7% (1.8–6.8), *p *= 0.0039, Fig. 2F and H) suggesting a more widespread failure of Jak/Stat signalling in γ-H2AX + CD8+ T-lymphocytes.

## Discussion

γ-H2AX expression in circulating lymphocytes, induced by DSBs and indicative of DDR activation, correlated with reduced telomere length in CD8+ T cells and fibrosis stage. The correlation between fibrosis stage and the small proportion of circulating γ-H2AX-positive lymphocytes was perhaps surprising and may reflect accelerated ageing in many tissues including the liver, or a more direct, potent effect mediated through the secretory phenotype of senescent cells. γ-H2AX expression was confined to T cells with an advanced cell-surface phenotype and associated with a skewed cytokine expression profile (increased IFN-γ with reduced IL-2) consistent with previous data in CD4+ T lymphocytes in HCV infection [23]. γ-H2AX + CD8+ T lymphocytes had impaired phosphorylation of STAT1 in response to exogenous IFN-α, which was not explained by altered expression of IFN-AR2 or IFN-AR1, or there was evidence that the failure to phosphorylate Jak1 and Tyk2 was associated with the intracellular apparatus of the IFN-α receptor. γ-H2AX + CD8+ T lymphocytes are more abundant within HCV-infected liver and had similar surface phenotype and functional deficits to circulating γ-H2AX + CD8+ T-lymphocytes.

There was an unexplained mechanistic association between telomere shortening, downstream signalling and the failure of γ-H2AX positive cells to respond to IFN-α by phosphorylation of STAT1. Possible explanations include a lesion of either Jak1 or Tyk2 [24], expression of the truncated isoform of IFN-AR2 [25] or suppression of signalling through the suppressor of cytokine signalling (SOCS) family of proteins [26].

Pre-translational processing involving mRNA splicing can result in expression of a truncated isoform of IFN-AR2, which suppresses signalling through the IFN-AR complex to intracellular second messengers [25]. Murine cell lines, infected with VZV, failed to suppress viral replication when signalling through the isoform with a truncated cytoplasmic tail, in contrast to full length IFN-AR2. Human cells normally express the truncated isoform at low levels compared to the full length biologically active form [27]. However, studies have demonstrated that other chronic viral infections modulate the frequency of IFN-AR splice isoforms [27,28].

Hepatitis D virus suppresses IFN-α signalling through inhibition of Tyk2 phosphorylation, preventing STAT1 phosphorylation and activation [24]. Our data precluded a similar mechanism, but there may be a failure of kinase function of the activated intracellular tail of the IFN-α receptor.

γ-H2AX expression often reflects the cellular response to shortened telomeres, but is also generated at the site of any break in double-stranded DNA. γ-H2AX expression is related to critical telomere shortening, but that is not the only explanation [10,11,29]. In cells exposed to increased DNA damage, through oncogene or oxidative stress, the direct correlation between γ-H2AX expression and telomere length can be lost. Senescence, however induced, leads to increased ROS production, leading to increased DSB foci within the cell [30]. Viruses, including EBV, induce γ-H2AX in infected cells [31]. Herpes virus γ-HV68 infection in a murine model led to γ-H2AX induction, which in turn increased viral replication [31]. Viruses induce cell-cycle arrest in order to generate a more favourable environment for replication. A direct effect of HCV on γ-H2AX expression in T cells is feasible, but was not investigated.

Chronic exposure of cells to IFN-β, but not IFN-α, led to irreversible p53-dependent cell-cycle arrest [32], whereby fibroblasts exposed to IFN-β for 6 days developed γ-H2AX expression, accumulation of mitochondrial-derived ROS and failure of proliferation. Perhaps the failure to demonstrate a stronger correlation between γ-H2AX and telomere length in patients with chronic HCV infection relates to chronic exposure of lymphocytes to IFN-α and subsequent expression of γ-H2AX. However, γ-H2AX + cells have increased expression of IFN-AR1, normally negatively regulated by IFN-α, which would suggest that these cells have not been stimulated through this pathway *in vivo*.

In a previous study, the degree of DNA damage in circulating lymphocytes in HCV-related, HBV-related and alcohol-related cirrhosis [33] correlated with the Child-Pugh score. Why circulating lymphocytes demonstrate DNA damage in patients with cirrhosis is unclear. Although we have excluded the possibility that γ-H2AX positive cells do not react to a single HLA-A2 restricted HCV epitope, it is possible that they might react to other epitopes in a non-HLA-A2 restricted fashion. However, we believe this unlikely given that the cells also do not react with the typically immunodominant A2-restricted pp65 epitope for CMV. Several previous studies have postulated the role of a bystander effect induced by chronic cytokine secretion in response to chronic viral infection leading to telomerase inhibition in the general, rather than antigen-specific, T-lymphocyte population [34]. It is plausible that γ-H2AX positive cells represent a multi-specific memory cell population that has re-circulated through the chronically inflamed liver in the context of HCV infection. Higher proportions of γ-H2AX positive cells in the liver would support this possibility. However, other groups have demonstrated that HCV infection is associated with a failure of maturation of non-HCV specific cells. Lucas *et al.* demonstrated that CMV-specific CD8+ T lymphocytes in HCV had fewer surface markers of an advanced differentiation state compared to non-HCV infected subjects [35].

Chronic HCV infection modulates the response to IFN-α through pSTAT1. HCV-NS5A prevents phosphorylation and nuclear translocation of pSTAT1 [18]. HCV-transfection of HuH7 cells leads to enhanced pSTAT1 degradation [36]. The N-terminal portion of HCV-core binds to the C-terminal portion of STAT1, blocking both homo- and hetero-dimerisation, preventing its intracellular actions [17]. HCV core protein reduced pSTAT1 in circulating T lymphocytes but increased pSTAT1 in B-lymphocytes HCV [37]. These findings may explain in part the increased EC50 for IFN-α in HCV infection, but not the very significant pathway defects seen in γ-H2AX + CD8+ T cells.

The failure to respond to IL-6 and IL-2 by phosphorylation of STAT1 and STAT5 respectively suggests a broader defect in senescent T lymphocytes in response to exogenous cytokine signals.

CD8+ γ-H2AX+ T lymphocytes have shortened telomeres, are highly differentiated, associated with severe fibrosis, are more frequent within the liver and although they have evidence of effector function (IFN-γ expression), do not activate Jak/Stat pathways in response to IFN-α, IL-2 or IL-6, perhaps explaining the failure of older patients and those with severe fibrosis to respond to treatment. Defining the cellular pathway linking advanced fibrosis, telomere shortening and failure to respond to IFN-α therapy could be important.

## Financial support

MH was supported by a Wellcome Trust Clinical Research Training Fellowship. Consumable expenses were supported by the Addenbrooke’s Hepatology Endowment Fund and the Adenbrooke’s Liver Transplant Association.

## Conflict of interest

The authors who have taken part in this study declare that they have nothing to disclose regarding funding or conflict of interest with respect to this manuscript.

## Figures and Tables

**Table 1:** **Demographic data of subjects in the four study groups**.

Significant values are showed in bold. ^†^Kruskal Wallis unless otherwise stated. ^1^Chi-squared.

**Table 2:** **Clinical and demographic factors associated with γ-H2AX expression on CD8+ T lymphocytes from HCV-RNA+ subjects (n = 109) by multiple linear regression**.

Significant values are showed in bold.

## Supplementary data

Supplementary Fig. 1 **Association of γ-H2AX expression on CD8+ T-lymphocytes with demographics and serostatus**. Panels A–C. Correlations between γ-H2AX expression on CD8+ T-lymphocytes and serum ALT (Panel A), Bilirubin (Panel B) or prothrombin time (Panel C) in peripheral blood from HCV RNA+ subjects (n = 109). Panels D through I. Relationship between γ-H2AX expression on CD8+ T-lymphocytes and serostatus or demographics in the entire cohort (n = 161). Association between γ-H2AX expression on CD8+ T-lymphocytes and age (Panel D), anti-HBcoreserostatus (Panel E) or BMI (Panel F). Panel G–I. Association between γ-H2AX expression on CD8+ T-lymphocytes and CMV serostatus (n = 161).Analysis by Mann Whitney U test. Panel H. Circulating CD8+ T-lymphocyte subsets, defined by CD27 and CD57 expression, in CMV seronegative (n = 38) and seropositive subjects (n = 43). Panel F. γ-H2AX expression on CD8 + CD27- T-lymphocytes from seronegative (n = 38) and seropositive subjects (n = 43).

Supplementary Fig. 2 **Cell surface markers of activation and inhibitory receptors on γ-H2AX+ CD8+ lymphocytes**. Cell surface expression of PD-1 (Panels A&B), CD38 (Panel C), CD69 (Panel D) and Tim3 (Panel E) on γ-H2AX-CD8+ (red lines) and γ-H2AX-CD8+ (blue lines) T-lymphocytes from subjects with viraemic HCV infection (n = 3).

Supplementary Fig. 3 **Antigen specificity of γ-H2AX+ CD8+ lymphocytes by class 1 pentamer analysis**. Three HLA-A2 positive subjects with HCV viraemia and seropositive for CMV were studied for co-expression of γ-H2AX and pentamers for HCV-NS3 KLVALGINAV (upper panels) or CMV-pp65 NLVPMVATV (lower panels) on circulating CD8+ T-lymphocytes.

Supplementary Fig. 4 **Failure of CD8+ γ-H2AX+ cells to phosphorylate Stat1 does not relate to Stat1 expression or a failure to phosphorylate Tyk2 or Jak1**. Panel A & B. Stat1 expression of CD8+ T-lymphocytes from subjects with viraemic HCV infection (n = 15); (A) example flow-cytometric plot demonstrating co-expression of γ-H2AX and whole Stat1; (B) stat1 expression in whole and γ-H2AX+ lymphocytes. Analysis by Wilcoxon signed rank test. Phosphorylation of Tyk2 (Panel C) and Jak1 (Panel D) on whole CD8+ and γ-H2AX+ CD8+ lymphocytes from viraemic HCV infected subjects (n = 9). Panels C&D demonstrate proportion of whole CD8+ and γ-H2AX+ CD8+ lymphocytes expressing phospho-Tyk2 (C) and phospho-Jak1 (D) after incubation with 1000 iu/ml IFN-α2b. Stats by Wilcoxon signed rank test.
