# Supplementary figures and images for: γ-H2AX + CD8+ T lymphocytes cannot respond to IFN-α, IL-2 or IL-6 in chronic hepatitis C virus infection
Source: J Hepatol. 2013 May;58(5):868–74. doi: 10.1016/j.jhep.2012.12.009 (PMC3625113; doi:10.1016/j.jhep.2012.12.009)

## Slide 1
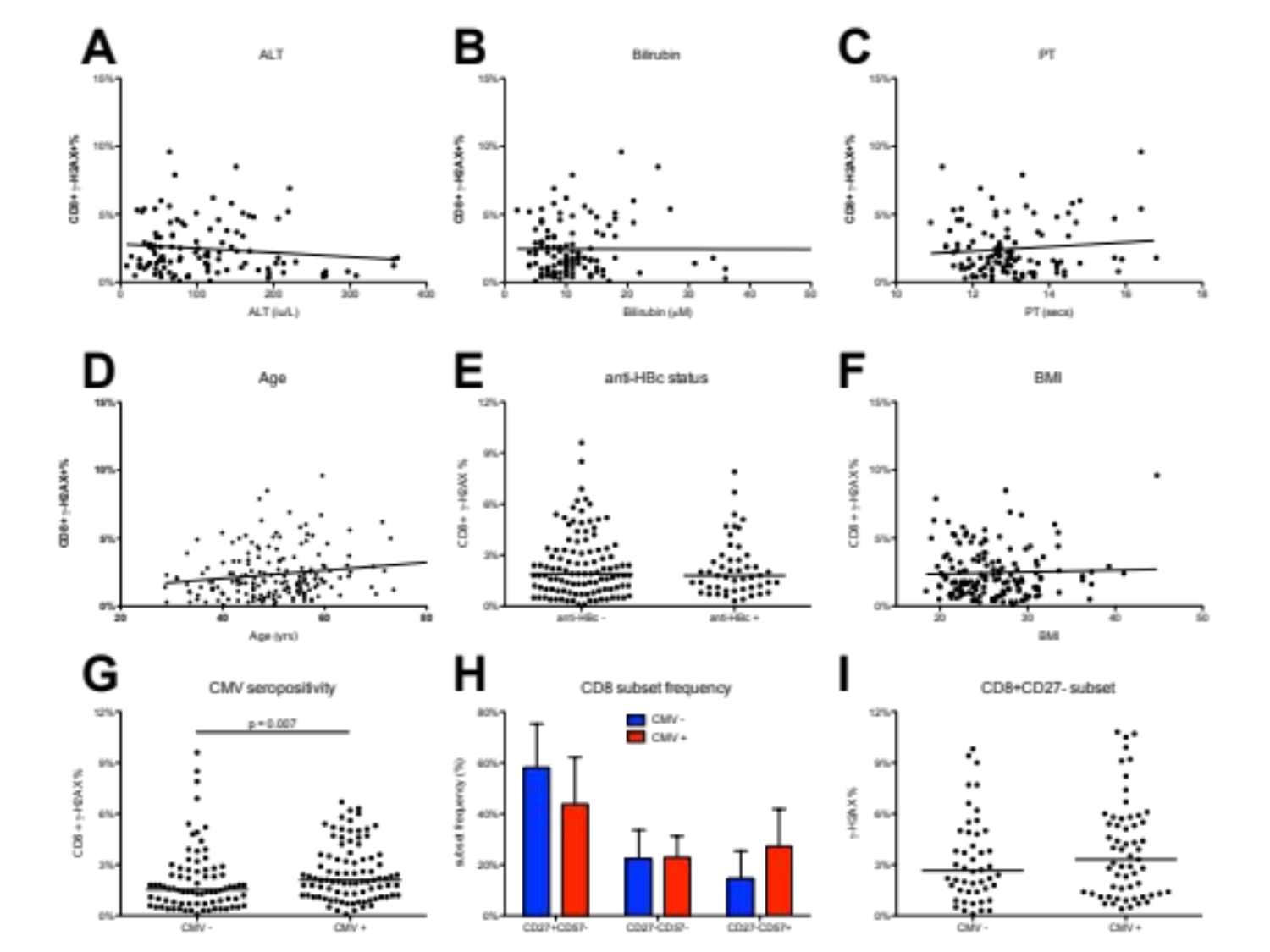

Supplement: Supplementary Fig. 1 — Association of γ-H2AX expression on CD8+ T-lymphocytes with demographics and serostatus. Panels A–C. Correlations between γ-H2AX expression on CD8+ T-lymphocytes and serum ALT (Panel A), Bilirubin (Panel B) or prothrombin time (Panel C) in peripheral blood from HCV RNA+ subjects (n = 109). Panels D through I. Relationship between γ-H2AX expression on CD8+ T-lymphocytes and serostatus or demographics in the entire cohort (n = 161). Association between γ-H2AX expression on CD8+ T-lymphocytes and age (Panel D), anti-HBcoreserostatus (Panel E) or BMI (Panel F). Panel G–I. Association between γ-H2AX expression on CD8+ T-lymphocytes and CMV serostatus (n = 161).Analysis by Mann Whitney U test. Panel H. Circulating CD8+ T-lymphocyte subsets, defined by CD27 and CD57 expression, in CMV seronegative (n = 38) and seropositive subjects (n = 43). Panel F. γ-H2AX expression on CD8 + CD27- T-lymphocytes from seronegative (n = 38) and seropositive subjects (n = 43). [file mmc1.pptx]

## Slide 1
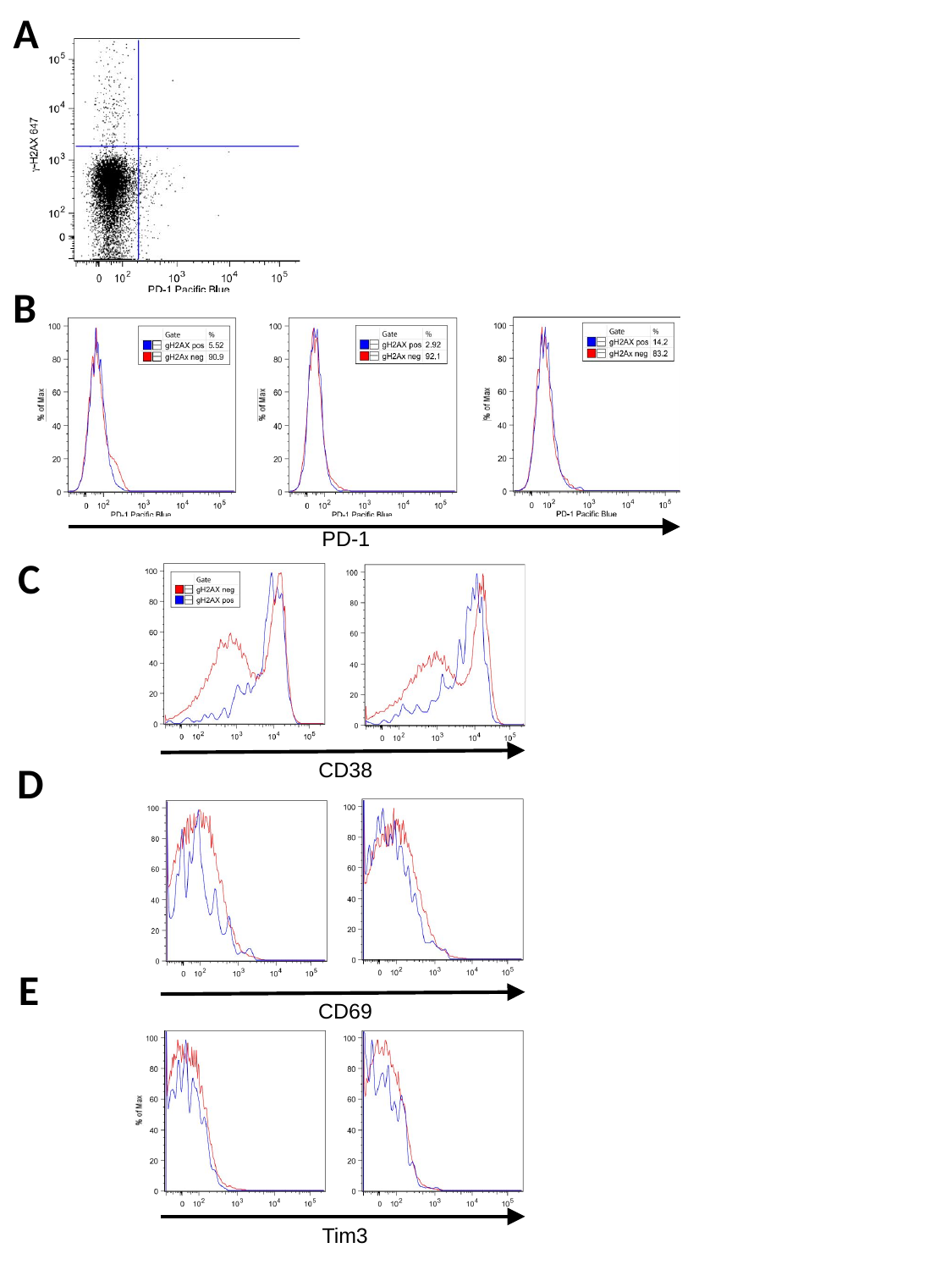

A
B
PD-1
C
CD38
D
E
CD69
Tim3

Supplement: Supplementary Fig. 2 — Cell surface markers of activation and inhibitory receptors on γ-H2AX+ CD8+ lymphocytes. Cell surface expression of PD-1 (Panels A&B), CD38 (Panel C), CD69 (Panel D) and Tim3 (Panel E) on γ-H2AX-CD8+ (red lines) and γ-H2AX-CD8+ (blue lines) T-lymphocytes from subjects with viraemic HCV infection (n = 3). [file mmc2.pptx]

## Slide 1
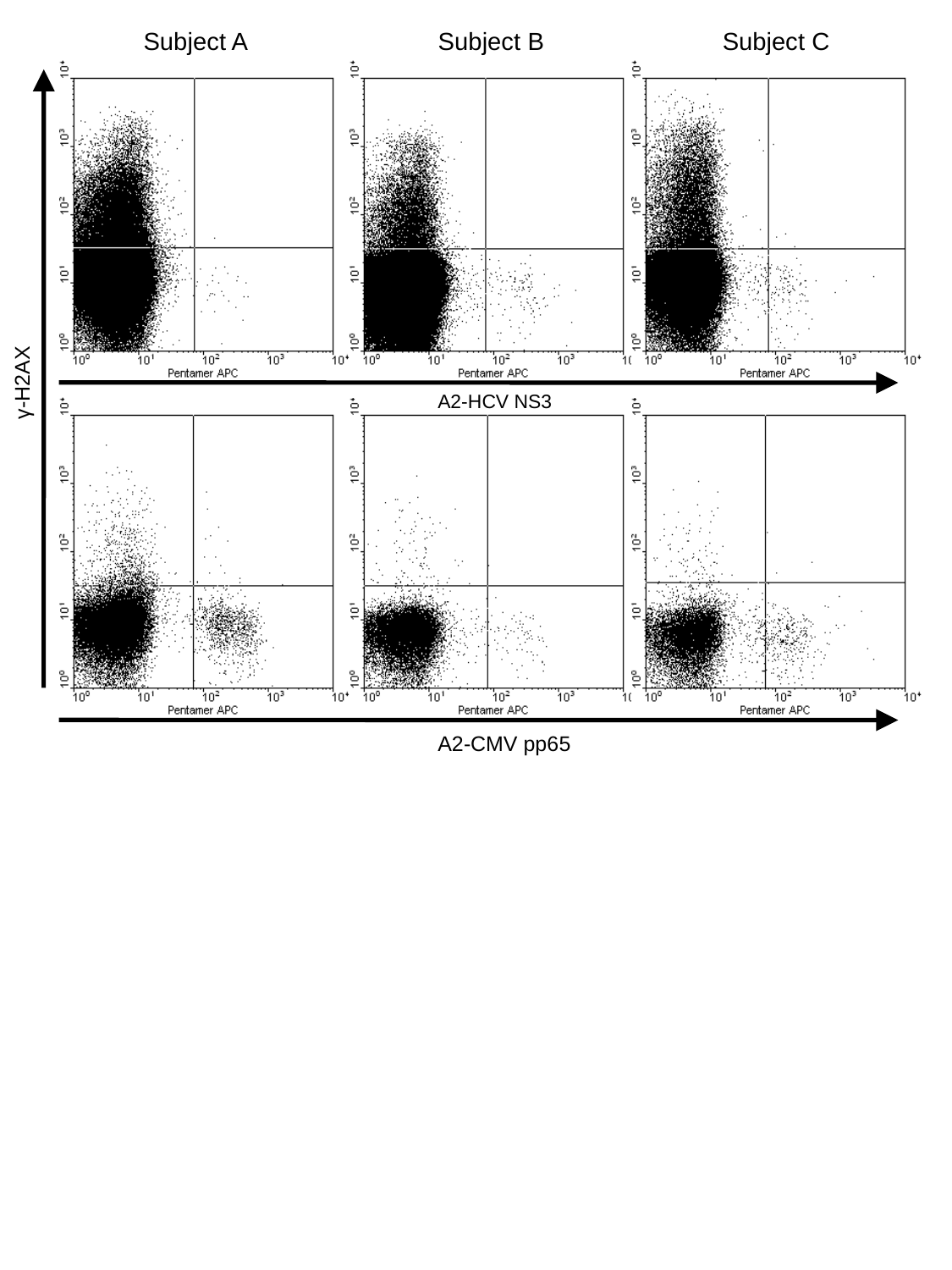

Subject A
Subject B
Subject C
γ-H2AX
A2-HCV NS3
A2-CMV pp65

Supplement: Supplementary Fig. 3 — Antigen specificity of γ-H2AX+ CD8+ lymphocytes by class 1 pentamer analysis. Three HLA-A2 positive subjects with HCV viraemia and seropositive for CMV were studied for co-expression of γ-H2AX and pentamers for HCV-NS3 KLVALGINAV (upper panels) or CMV-pp65 NLVPMVATV (lower panels) on circulating CD8+ T-lymphocytes. [file mmc3.pptx]

## Slide 1
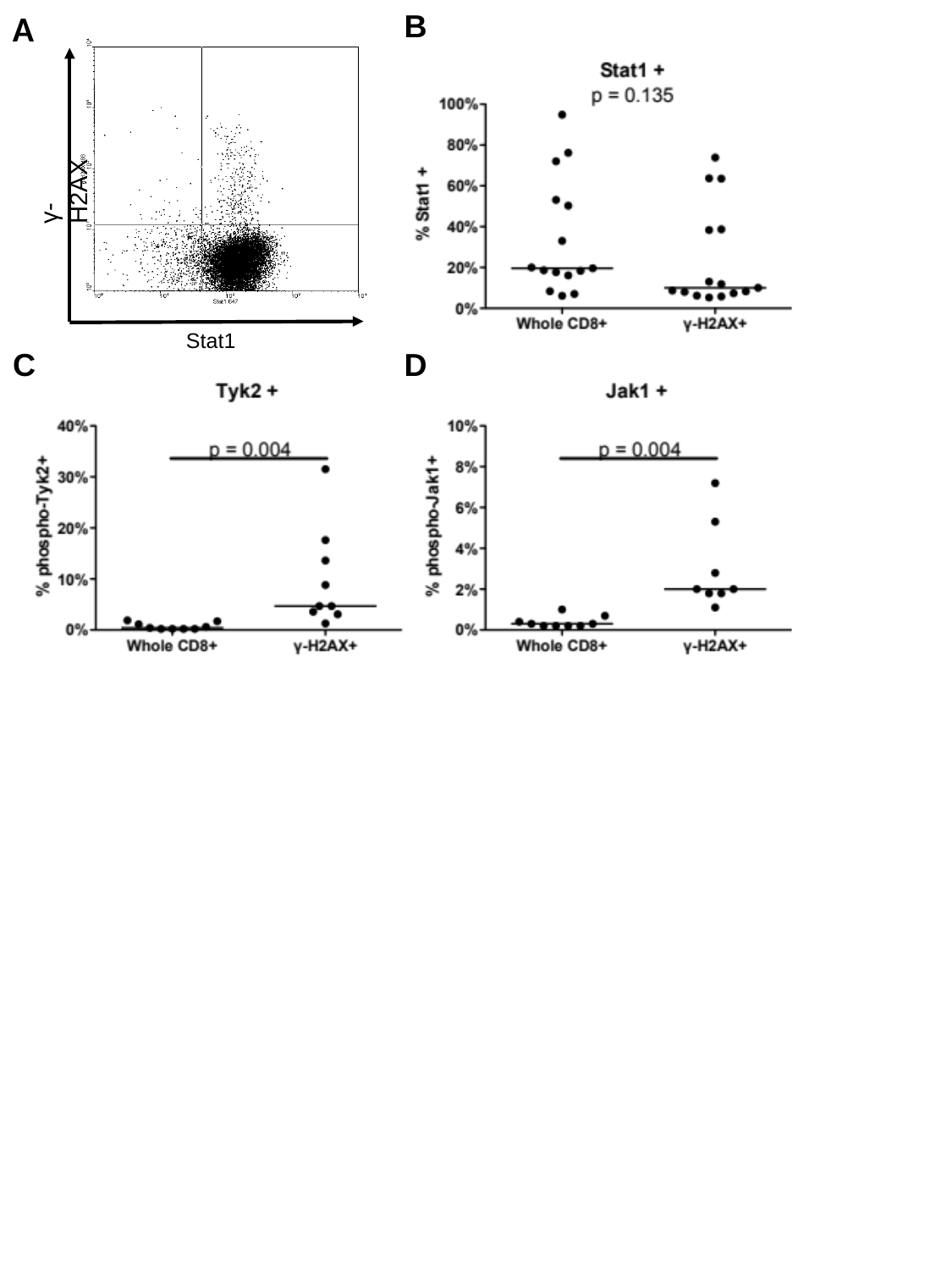

B
A
γ-H2AX
Stat1
C
D

Supplement: Supplementary Fig. 4 — Failure of CD8+ γ-H2AX+ cells to phosphorylate Stat1 does not relate to Stat1 expression or a failure to phosphorylate Tyk2 or Jak1. Panel A & B. Stat1 expression of CD8+ T-lymphocytes from subjects with viraemic HCV infection (n = 15); (A) example flow-cytometric plot demonstrating co-expression of γ-H2AX and whole Stat1; (B) stat1 expression in whole and γ-H2AX+ lymphocytes. Analysis by Wilcoxon signed rank test. Phosphorylation of Tyk2 (Panel C) and Jak1 (Panel D) on whole CD8+ and γ-H2AX+ CD8+ lymphocytes from viraemic HCV infected subjects (n = 9). Panels C&D demonstrate proportion of whole CD8+ and γ-H2AX+ CD8+ lymphocytes expressing phospho-Tyk2 (C) and phospho-Jak1 (D) after incubation with 1000 iu/ml IFN-α2b. Stats by Wilcoxon signed rank test. [file mmc4.pptx]
