# Supplementary material for: γ-H2AX + CD8+ T lymphocytes cannot respond to IFN-α, IL-2 or IL-6 in chronic hepatitis C virus infection
Source: J Hepatol. 2013 May;58(5):868–74. doi: 10.1016/j.jhep.2012.12.009 (PMC3625113; doi:10.1016/j.jhep.2012.12.009)
Supplement: Supplementary Materials and methods [file mmc5.docx]

**Supplementary Materials and methods**

*Liver biopsy*

Circulating and intrahepatic lymphocytes were obtained simultaneously from HCV-infected patients undergoing percutaneous liver biopsy. Liver biopsy was performed with a 1.9mm diameter Menghini needle (Steriseal). Liver tissue was disaggregated mechanically in RPMI-1640, passed through a 70µm nylon filter before layering over Lymphoprep.

*Immunoblotting*

Magnetic bead separated cells were lysed for 5 minutes in 50mM HEPES, 100mM NaCl, 1% triton X-100, 2mM EDTA pH 7.7. For each gel, 5 μg of protein was loaded per lane on 12.5% SDS PAGE gels, transferred to PVDF, blocked in Tris buffered saline with 1% Tween and 5% nonfat dry milk for 30 minutes and incubated overnight with primary antibodies to γ-H2AX (ser-139) (Cell Signaling) or β-Actin (Sigma). Blots were then washed and incubated for 1 hour in horseradish peroxidase conjugated secondary antibody and developed using enhanced chemiluminescence (GE Healthcare, UK).

*Flow cytometry*

PBMCs were stained with combinations of CD8-biotin, CD8 PE-Cy5 (Ebioscience), CD8-Qdot605 (Invitrogen), CD27-APC (BD), CD38-APC (Ebioscience), CD45RO-biotin (Ebioscience), CD45RO-FITC (Dako), CD57-biotin (BD), CD69-APC (Ebioscience), PD-1-Pacific Blue (Ebioscience), Tim3-APC (Ebioscience) before fixation in CALTAG medium A. Cells were permeabilised in ice-cold 90% methanol (VWR) and stained with combinations of Alexa Fluor (AF) 488 or AF647 conjugated anti-γ-H2AX (ser-139), AF647 conjugated anti-phospho-p53 (ser-15) AF488-conjugated anti-pStat1 (Y701) or AF488 conjugated anti-pStat5 (Y694) (all Cell Signaling), FITC-conjugated IFN-γ, FITC-conjugated IL2, PE-conjugated anti-human Ki67 (BD) or appropriate isotype controls.

For phospho-Tyk2 and -Jak1 staining rabbit anti-phospho-Jak1 or rabbit anti-phospho-Tyk2 (Santa-Cruz) and mouse anti-γ-H2AX (Abcam) were used. Cells were then washed twice, blocked in goat serum, prior to incubation with goat anti-rabbit Alexa Fluor-488 and goat anti-mouse Alexa-Fluor 647 (Invitrogen).
